# The 2006 Automated Function Prediction Meeting

**DOI:** 10.1186/1471-2105-8-S4-S1

**Published:** 2007-05-22

**Authors:** Ana PC Rodrigues, Barry J Grant, Adam Godzik, Iddo Friedberg

**Affiliations:** 1 Burnham Institute for Medical Research, 10901 N. Torrey Pines Rd., La Jolla, CA 92037 USA; 2 Department of Chemistry and Biochemistry, University of California, San Diego, La Jolla, CA 92093, USA; 3 Center for Research in Biological Systems (CRBS), University of California, San Diego, 9500 Gilman Drive La Jolla, MC 0446 CA 92093, USA

Genomic scale projects have compounded the need for rapid and reliable functional annotation methods. Traditional experimental approaches have become outpaced resulting in an ever-increasing proportion of missing annotations. Computational approaches, including those based on sequence, expression, interaction and tertiary structure, have the potential to impact the growing annotation deficit.

Despite a recent increase in the number and variety of prediction methods, the computational annotation of protein function remains difficult. This stems from a combination of issues such as the inherent limitations of current tools and databases, the difficulty of assessing the predictive power of different methods and more fundamental problems related to the ambiguity of the definition of function itself.

These and related themes were addressed at the second Automated Function Prediction (AFP) conference, which took place at the University of California, San Diego campus in late August 2006. AFP2006 attracted more than 100 participants and extended over three days with 8 keynote presentations, 19 contributed talks, 20 posters and a panel discussion. A broad range of function prediction methods were presented, focusing on the development of new techniques as well as the thorough utilization of the spectrum of data being produced by genomics and post-genomics research. The importance of standardized functional dictionaries capable of incorporating a range of function definitions, such as those implemented by the popular Gene Ontology (GO) project [1], the Structural Classification of Proteins (SCOP) [2] and the Enzyme Classification (EC) system [3,4] was made apparent throughout the meeting, by their pervasive usage in the implementation and assessment of protein function prediction methods. Discussions of the need for reliability indicators and blind validations of the various methods led to consensus agreement on the value of conducting a community wide assessment of protein function experiment (*a-là *CASP [5] for assessment of protein structure predictions and CAPRI [6] for the assessment of protein interactions). For this supplement we have chosen nine studies presented at AFP 2006 to be published as full-length articles.

Most computational methods for functional annotation rely on the transfer of knowledge accumulated in sequence and structure databases to related proteins. These methods can be distinguished by the manner in which the 'relatedness' of proteins is defined: some employ sequence similarity and structure similarity measures, others gene order conservation, co-occurrence across genomes and even shared interaction partners. The first seven papers in this supplement describe new developments for each of these methods.

Homology-based methods are underpinned by the conservation of functionally important residues, and employ sequence and/or structure similarity to identify functionally related proteins.

Melvin and colleagues [7] describe SVM-Fold, a new method for remote homology detection and fold recognition. The method employs a support vector machine algorithm with kernels based on PSI-BLAST [8] profiles, as described in [9]. A novel multi-class classification algorithm, termed adaptive codes, is employed to exploit the hierarchical information contained within the SCOP [2] database. The authors show that, in comparison with PSI-BLAST and their previously described algorithm, SVM-fold improves remote homology detection and significantly improves fold recognition.

In related work, Audit and colleagues [10] detail the application of their previously described probabilistic framework for homology-based annotations [11] to the ENZYME database [4]. This framework combines the pairwise similarity scores between query sequence and all members of a functional class to measure the relationship between protein and class. It then employs a Bayesian procedure to compute the likelihood that a new sequence belongs to that functional class. ENZYME re-annotations are thus assigned a probability value measuring the reliability of each prediction. Among the different classes, error rates range from 0 to 13.6%, mostly reflecting the inability of sequence similarity search procedures to detect substrate specificity.

Marti-Renom and colleagues [12] introduce two new programs, AnnoLite and AnnoLyze, which add functional content to the previously established DBAli database of protein structure alignments [13]. The AnnoLite program utilizes structural alignments to transfer functional annotations using the recognized vocabularies of SCOP [2], CATH [14], EC [3,4], GO [1], InterPro [15] and Pfam [16]. The AnnoLyze program utilizes structural alignments to transfer ligand binding site and domain interaction patch annotations from LigBase [17] and PIBASE [18], respectively. Importantly, for both tools, the authors define annotation specific cutoffs of sequence and structure similarity for confident transfer of annotations between proteins.

Henschel and colleagues [19] present a collection of hidden Markov models of protein-binding and ligand-binding interfaces. The models are generated using a multiple-motif approach to represent binding sites as a collection of small HMMs, each derived from sequence segments that constitute structural features of the interaction site. The authors use cross-validation and comparison to literature-curated interactions to show that a significant number of their protein-protein interaction models can be used to recognize protein-protein interaction sites. In addition, they validate the protein-ligand interaction models through comparison with PROSITE motifs [20,21] associated with ligand binding sites.

Genome context analysis methods are based on short-range genome co-linearity and conservation of gene regulation, and use gene order and localization to identify functionally related proteins. Li and colleagues [22] describe SynFPS, a new method that uses genomic context to predict function. SynFPS differentiates itself from other methods by its ability to detect gene correspondence among genomes of weakly related organisms, thus eliminating the requirement of prior knowledge of the relationship among them. This is achieved through genome clustering based on gene distribution, followed by support vector machine training for function prediction. The system is shown to be particularly effective in the analysis of bacteriophage genomes, where the phylogenetic relationship among organisms is far from established.

Proteins with similar functions are often observed to co-occur across genomes and thus possess similar phylogenetic profiles. Cokus and colleagues [23] define a new heuristic for the application of phylogenetic profile analysis that accounts for the relationship among organisms in a computationally efficient manner. This is achieved through an all-versus-all comparison of phylogenetic profiles and the subsequent re-ordering of those profiles according to the established relationships. The authors show that accounting for the number of runs, or consecutive matches, between ordered profiles improves the identification of functionally related proteins, by distinguishing conservation within closely related organisms from conservation across more divergent species.

Protein-protein interaction based methods rely on the emerging protein-protein interaction datasets and exploit interaction partners to identify functionally related proteins. Chua and colleagues [24] employ the previously described FS-Weighted Averaging method [25], which makes functional inferences based on indirect interaction partners and topological weighting, to annotate seven genomes from a diverse range of organisms with GO terms [1] from all three ontologies. Despite the different numbers and types of protein-protein interaction datasets available for each of the genomes, the annotations are shown to be robust against noisy data and complementary to homology based methods.

The remaining two papers chosen for this supplement do not rely on annotation transfer from related proteins, but instead tackle the difficult question of *de novo *identification of ligand binding sites in protein structures. This form of annotation has assumed greater emphasis as the majority of structures solved by structural genomics projects are of unknown function and bear no sequence or structure based similarity to any proteins that have a known function.

Xie and Bourne [26] describe a new algorithm for the prediction of ligand-binding sites based on a simplified shape description of protein structure. The method partitions protein space with two boundaries: an environment boundary, containing both protein and potential ligand binding pockets, and a protein boundary. Clusters of atoms at the intersection of these boundaries are identified as potential binding sites and their distance and orientation in relation to the environment boundary are used to compute a novel measure, termed geometric potential. The authors show that the geometric potential can be used to distinguish ligand binding from non-ligand binding sites, is minimally affected by conformational changes and is sufficiently fast to be applied in large-scale calculations.

In related work, Yoon and colleagues [27] report the extension of the FEATURE method [28-32] to enable the discovery of unknown functional sites in protein structures. FEATURE vectors describe microenvironments around active sites and binding sites of proteins as normalized counts of physical and chemical properties within sets of concentric shells. The authors show that *k*-means clustering of these environments, using a weighted version of the Hamming distance between vectors, enables the discovery of microenvironment clusters highly enriched with known functional sites. Analysis of such sites will allow the calibration of size and inter-cluster distances, thus enabling the discovery of novel functional sites.

In the introduction to his keynote presentation, Christos Ouzounis (CERTH) commented: "I have been trying to escape this field because it is so hard... but it keeps haunting us, and [thus we] keep revisiting the problems [it presents]". Function prediction is indeed a challenging endeavor that is further hampered by the lack of a standard assessment framework [5,33]. It was inspiring to see that a vibrant community of researchers from varied backgrounds, reflected in the variety and scope of papers showcased in this supplement, is focusing on this important problem. We hope to see this trend continue for the third annual AFP meeting, which will be held in Vienna Austria, July 19 to 20 of 2007.

For further information and updates on AFP meetings see: 
